# Epicardial Fat Thickness as Cardiovascular Risk Factor and Therapeutic Target in Patients with Rheumatoid Arthritis Treated with Biological and Nonbiological Therapies

**DOI:** 10.1155/2014/782850

**Published:** 2014-12-10

**Authors:** Marcos M. Lima-Martínez, Ediris Campo, Johanmary Salazar, Mariela Paoli, Irama Maldonado, Carlota Acosta, Marianela Rodney, Miguel Contreras, Julio O. Cabrera-Rego, Gianluca Iacobellis

**Affiliations:** ^1^Division of Medical Physiology, Department of Physiological Sciences, University of Oriente, Ciudad Bolívar 8001, Venezuela; ^2^Endocrinology, Diabetes, Metabolism and Nutrition Unit, Orinoco Medical Center, Annex A. Siegert Avenue, Ciudad Bolívar 8001, Venezuela; ^3^Endocrinology Unit, University Hospital of Los Andes, Mérida 5101, Venezuela; ^4^Rheumatology Service, Ruiz y Paez University Hospital, Ciudad Bolívar 8001, Venezuela; ^5^Cardiology Service, Ruiz y Paez University Hospital, Ciudad Bolívar 8001, Venezuela; ^6^El Valle Medical Center, Nueva Esparta 6301, Venezuela; ^7^Intensive Coronary Care Unit, Hospital Manuel Fajardo, 10400 Havana, Cuba; ^8^Division of Endocrinology, Department of Medicine, University of Miami Miller School of Medicine, Miami, FL 33136, USA

## Abstract

Rheumatoid arthritis (RA) is a chronic inflammatory disease associated with high cardiovascular morbidity and mortality. Epicardial adipose tissue (EAT) thickness may act as a therapeutic target during treatments with drugs modulating the adipose tissue. We evaluate EAT thickness in RA patients treated with biological and nonbiological disease-modifying antirheumatic drugs (DMARDs). A cross-sectional study was conducted with a cohort of 34 female RA patients and 16 controls matched for age and body mass index (BMI). Plasma glucose, basal insulin, plasma lipids, and high-sensitivity C-reactive protein (hs-CRP) were assessed. EAT thickness and left ventricular mass (LVM) were measured by echocardiography. No significant differences in waist circumference (WC), blood pressure, fasting blood glucose, basal insulin, and lipid parameters were found between the groups. The control group showed lower concentrations (*P* = 0.033) of hs-CRP and LVM (*P* = 0.0001) than those of the two RA groups. Patients treated with TNF-*α* inhibitors showed significantly lower EAT thickness than those treated with nonbiological DMARDs (8.56 ± 1.90 mm versus 9.71 ± 1.45 mm; *P* = 0.04). Women with no RA revealed reduced EAT thickness (5.39 ± 1.52 mm) as compared to all RA patients (*P* = 0.001). Results suggest that RA patients have greater EAT thickness than controls regardless of BMI and WC.

## 1. Introduction

Rheumatoid arthritis (RA) is a chronic inflammatory disease associated with high cardiovascular morbidity and mortality [1]. Traditional risk factors along with inflammation and autoimmunity contribute to the development of coronary artery disease in RA patients [1]. Furthermore, a growing body of evidence has proved that these subjects present early alterations in some subclinical atherosclerosis markers [2, 3].

Tumor necrosis factor-alpha (TNF-*α*) is the key cytokine in RA inflammatory processes. Several clinical studies have proved that TNF-*α* inhibitors are effective in reducing the clinical signs of inflammation in RA patients whose treatment with nonbiological disease-modifying antirheumatic drugs (DMARDs) has been unsatisfactory [4, 5]. An additional benefit of the treatment with TNF-*α* inhibitors is the reduction in the risk of cardiovascular events [6].

Epicardial adipose tissue (EAT) thickness has recently emerged as new marker of cardiometabolic risk [7]. Clinically, the thickness of epicardial fat can be easily and accurately measured [8]. Epicardial fat thickness can serve as marker of visceral adiposity and visceral fat changes during treatments with drugs targeting the fat [9, 10]. A meta-analysis conducted on 9 studies showed that EAT thickness was significantly higher in patients with metabolic syndrome (MS) than in those without it [11].

Recently, Ormseth et al. [12] demonstrated that EAT volume correlates with the components of MS in subjects with RA. However, it is unclear whether the interplay between EAT and RA is independent of MS. In addition, given its intrinsic inflammatory status, EAT displays the potential to serve as therapeutic target in patients with RA. Nevertheless, to date there is no study that evaluates the effect that immunological therapy based on TNF-*α* inhibitors has on EAT thickness in RA patients. In this study, we sought to evaluate epicardial fat thickness in RA patients treated with biological and nonbiological DMARDs.

## 2. Methodology

### 2.1. Study Design

A cross-sectional study was designed from a sample of RA female patients aged 18 to 65 years, evaluated at the Rheumatology Service of the Ruiz y Paez University Hospital in Ciudad Bolivar, Venezuela. The study was conducted following the recommendations of the Declaration of Helsinki and was approved by the Ethics Committee of our institution. All patients gave their informed consent before the beginning of the study.

### 2.2. Inclusion Criteria

Subjects with RA and a score ≥6/10 based on current criteria for the diagnosis of RA from the American College of Rheumatology (ACR) and the European League against Rheumatism (EULAR) [13] were included. All subjects received at least six months of treatment with either of the biologic DMARDs based on the use of TNF-*α* inhibitors or nonbiologic DMARDs. These female patients were compared with a group of women without RA (control) matched for age and body mass index (BMI).

### 2.3. Exclusion Criteria

Patients were excluded if they had a previous history of ischemic heart disease, cerebrovascular disease, high blood pressure, or receiving dialysis because of chronic kidney disease, primary hyperlipidemia, and endocrinopathies such as diabetes mellitus, hypothyroidism, Cushing syndrome, acromegaly, or any other comorbidity capable of affecting the metabolic variables.

### 2.4. Clinical Evaluation

A brief anamnesis was conducted to obtain demographic data, such as age, place of birth, duration of disease, and treatment history. Weight and height data were collected while the subjects were fasting and wearing only their underwear. Body mass index (BMI) was calculated as body weight divided by height squared in meters. Waist circumference (WC) was measured mid-waist between the lower margin of the rib cage and the iliac crest, with the patient in a standing position with minimal respiration, and expressed in centimeters. Blood pressure was measured in the right arm, after a 10-minute rest in a sitting position, by the auscultatory method, with a standard mercury sphygmomanometer.

The disease activity score (DAS 28) was assessed by total joint count (28 joints).

### 2.5. Biochemical Variables

A blood sample was taken in the morning after 8 hours fasting from the antecubital vein to determine serum glucose and blood lipids (total cholesterol, triglycerides, and HDL-C) by enzymatic methods. LDL-C was estimated through Friedewald's equation, where LDL-C = total cholesterol − [HDL-C + (triglycerides/5)]. Non-HDL-C was obtained by subtracting HDL-C from total cholesterol, and Tg/HDL ratio was determined by dividing triglycerides plasma concentration by HDL-C.

### 2.6. Echocardiographic Parameters

A transthoracic two-dimensional (2D) echocardiography examination was performed on each subject, using the Mylab 50 Xvision Esaote (Genoa, Italy) scanner as standard technique with patients in the left lateral decubitus position by an echocardiographist blinded to RA diagnosis, as well as clinical data and therapy. Epicardial fat thickness was measured according to the method first described and validated by Iacobellis et al. [14].

Epicardial fat was identified as the echo-free space between the outer wall of the myocardium and the visceral layer of pericardium. Epicardial fat thickness was measured in the parasternal long-axis view, perpendicularly on the free wall of the right ventricle at end-systole in three cardiac cycles. Maximum epicardial fat thickness was measured at the point on the free wall of the right ventricle along the midline of the ultrasound beam, perpendicular to the aortic annulus, used as anatomical landmark for this view. The average value of three cardiac cycles was considered [8].

Left ventricular mass (LVM) was similarly determined by parasternal long-axis view using an anatomically validated formula of Devereux et al. [15], subsequently indexed to the patient's body surface area.

### 2.7. Statistical Analysis

Continuous variables are expressed as mean ± SD. Mean differences between continuous variables in the three groups (control, nonbiological, and biological DMARDs) were determined by a variance analysis (ANOVA) and LSD as a* post hoc* test when variables showed a normal distribution and Kruskal-Wallis test when distribution differed from the norm (systolic and diastolic blood pressure). A Student's *t*-test for independent data assessed the mean difference of normally distributed continuous variables between the two groups with RA (nonbiological and biological DMARDs). Pearson's correlation matrix was performed, as well as a multiple linear regression analysis, taking epicardial fat as the dependent variable in order to determine which variable had more weight upon it. SPSS 20.0 for Windows was used for the statistical analysis, and a value of *P* ≤ 0.05 was considered statistically significant.

## 3. Results

Table 1 shows the patients' anthropometric and clinical data. Thirty-four RA subjects were studied, 18 receiving monotherapy with biological DMARDs based on TNF-*α* inhibitor and 16 with nonbiological DMARDs (9 patients were on methotrexate, 4 on chloroquine, and 3 on leflunomide). The group treated with nonbiological DMARDs had an average age of 51.31 ± 6.70 years and a BMI of 28.65 ± 7.09 Kg/m^2^. On the other hand, the group treated with biological DMARDs had an average age of 52.05 ± 8.26 years and a BMI of 28.20 ± 8.19 Kg/m^2^. The control group comprised 16 women without RA, with an average age of 51.81 ± 9.75 years and a BMI of 29.48 ± 7.35 Kg/m^2^. There were no significant differences between the groups regarding BMI, WC, and systolic (SBP) and diastolic (DBP) blood pressure. Similarly, significant differences were not found in RA duration of patients treated with nonbiological DMARDs (15.93 ± 9.27 years) in comparison with those treated with biological DMARDs (11.22 ± 7.93 years). Also, there were no differences in the number of patients treated with glucocorticoids in both groups (nonbiological DMARDs 13/16 versus biological DMARDs 12/18).

When comparing the biochemical variables among the groups (Table 2), no significant differences were observed in fasting blood glucose, basal insulin, total cholesterol, HDL-C, LDL-C, non-HDL-C, triglycerides, and Tg/HDL-C ratio. However, high-sensitivity C-reactive protein (hs-CRP) was significantly increased in both nonbiological and biological DMARDs groups when compared to the control group (*P* = 0.033). Also, LVM in the control group was lower than those of the two groups with RA (*P* = 0.0001).

Table 3 shows RA patients' acute phase reactants and activity indicators, where it was observed that patients treated with biological DMARDs showed lower levels of erythrocyte sedimentation rate (ESR), rheumatoid factor (RF), and disease activity (DAS28) when compared to those treated with nonbiological DMARDs. However, these differences were not significant.

**Figure 1:**
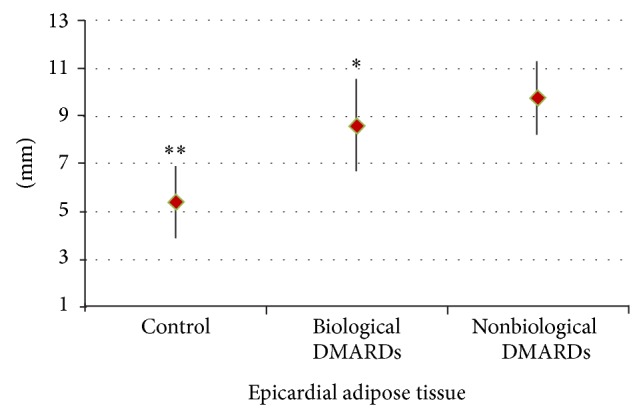
Epicardial adipose tissue thickness in the control group (5.39 ± 1.52 mm), in RA patients treated with biological DMARDs (8.56 ± 1.90 mm), and in RA patients treated with nonbiological DMARDs (9.71 ± 1.45 mm). ^*^ *P* = 0.04 versus RA nonbiological DMARDs. ^**^ *P* = 0.001 versus biological and nonbiological RA DMARDs.

Figure 1 shows that RA patients treated with biological DMARDs had a significantly lower EAT thickness than those treated with nonbiological DMARDs (8.56 ± 1.90 mm versus 9.71 ± 1.45 mm; *P* = 0.04), and women without RA had the lowest epicardial fat thickness (5.39 ± 1.52 mm) compared to all patients with RA (*P* = 0.001).

**Figure 2:**
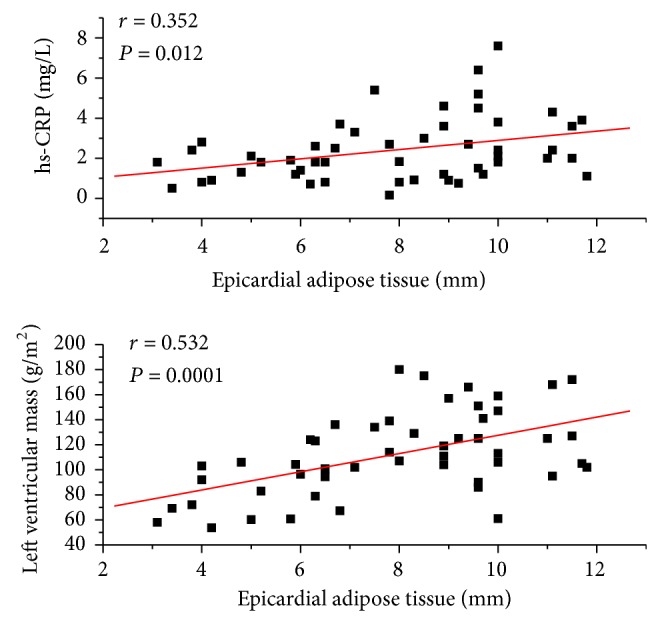
Correlation of epicardial adipose tissue thickness with plasma concentration of high-sensitivity C-reactive protein (hs-CRP) and left ventricular mass.

Epicardial fat thickness showed a significant and positive correlation with both hs-CRP plasma concentration (*r* = 0.353; *P* = 0.012) and LVM (*r* = 0.532; *P* = 0.0001) (Figure 2). There was no correlation of EAT with BMI, WC, SBP, and DBP.

A multivariate linear regression analysis was conducted (Table 4) to determine which variables exerted greater influence on EAT thickness as a dependent variable in the sample studied. Variables hs-CRP and LVM lost their statistical significance, whereas the presence or not of RA was very significant (*P* = 0.0001), this variable being the one having the most influence on epicardial fat, with a square *R* of 0.595.

## 4. Discussion

An elevated cardiovascular mortality is reported in subjects with RA. However, it is unknown if this is due to traditional risk factors or the result of the inflammatory process underlying the disease [16].

The main findings of this study are as follows: (1) female patients with RA have a greater EAT thickness than those without RA and (2) patients treated with biological DMARDs have lower epicardial fat thickness than those treated with nonbiological DMARDs.

EAT is a surrogate marker of visceral adiposity, and it has been demonstrated that visceral fat can be an independent predictor of metabolic risk [17]. A growing body of evidence has indicated that EAT thickness is significantly associated with conventional anthropometric and clinical variables, such as BMI, WC, SBP, and DBP [18, 19].

We found that patients with RA showed greater EAT thickness, as compared with subjects without the disease, regardless of their BMI and WC. This finding is of great importance, because it rules out the confounding effect of obesity and MS on the interplay between EAT and RA. EAT is higher in subjects with RA, likely reflecting a higher visceral fat accumulation independent of MS and obesity by itself. Remarkably all factors related to MS were similar between the control group and biological and nonbiological DMARDs groups.

The higher visceral adiposity, here reflected by higher EAT, could be related to the use of glucocorticoids by RA patients, as these drugs promote large deposition of visceral fat [20]. Interestingly, the articular and extra-articular changes associated with RA produce alterations in body fat distribution, which includes the so-called “cachectic obesity” characterized by loss of muscle tissue and significant fat gain [21]. Thus, it can be assumed that EAT thickness constitutes a better marker of visceral adiposity when compared with BMI and WC.

As far as we know, this is the first study where the effect of biological therapy on EAT thickness has been evaluated, and there are few studies that have determined the effect of TNF-*α* inhibitors on the overall adipose tissue. Šenolt et al. [22] proved that treatment with etanercept, a TNF-*α* inhibitor, is associated with an increase in leptin expression and lowering of adiponectin levels in the subcutaneous adipose tissue of subjects with RA, and Renzo et al. [23] observed, in psoriasis vulgaris and psoriatic arthritis patients treated with TNF-*α* inhibitors, an 8.6% and an 8.9% gain in fat mass, respectively. It is worth highlighting that this study did not differentiate between subcutaneous and visceral adipose tissues, which differ in both embryologic origin and metabolic functions [24, 25]. Interestingly, it has been demonstrated that production of inflammatory cytokines and infiltration by inflammatory cells are greater in EAT than in the subcutaneous adipose tissue, and a strong correlation has been observed between plasma TNF-*α* concentrations and the number of macrophages that infiltrate EAT [26, 27].

Recently, macrophages have received special interest as mediators of inflammatory response and insulin resistance in the adipocyte. It has been demonstrated that macrophages, through the production of TNF-*α* and the subsequent activation of nuclear factor kappa-light-chain-enhancer of activated B cells (NF-*κ*B), reduce the sensitivity to insulin in the adipocyte through decrease of glucose transporter type 4 (GLUT-4) and insulin receptor substrate (IRS-1) expression [28, 29]. Likewise, this cytokine can inhibit the differentiation process from preadipocyte to adipocyte [29, 30], and treatment with anti-TNF-alpha monoclonal antibodies can partially revert such deleterious effects [28]. These findings suggest that the interaction between these cells causes a reduction in glucose transport in the adipocyte that can contribute to systemic insulin resistance. This hypothesis is supported by a recent study by Goldfine et al. [31] who demonstrated that the pharmacological treatment with an NF-*κ*B inhibitor has antihyperglycemic effects in subjects with type 2 diabetes mellitus. Thus, it is possible to suggest that TNF-*α* inhibitors are associated with a lesser EAT thickness due to the reduction in the inflammatory response mediated by this cytokine and the better insulin sensitivity at the adipocyte level.

EAT is a source of several inflammatory mediators, and there is evidence that demonstrates the role of inflammation in the development of atherosclerosis in subjects with RA [32]. This study found a significant correlation between EAT thickness and hs-CRP plasma concentration; however, in the patients with RA no correlation was evidenced with other markers related to the disease, similar to Lipson et al. [33], observed in patients with systemic lupus erythematosus. Similarly, no correlation was found between EAT thickness and DAS28. In this regard, it is necessary to highlight that hs-CRP and ESR reflect the inflammatory state at sampling and depend not only on the disease activity but also on the treatment and even on genetic variations. Previous studies in RA patients have demonstrated the lack of association between DAS28 and other markers of subclinical atherosclerosis [34]. It is possible that the lack of association among the variables is due to the intermediary role of the glucocorticoids, since patients with more disease activity receive greater doses of these drugs. Additionally, the relatively low number of patients is a statistical limitation that could influence this result.

We found a significant correlation between LVM and EAT in our patients with RA, regardless of the treatment. Left ventricle hypertrophy is an independent cardiovascular risk factor, and an increase in LVM has been reported in subjects with RA [35]. This study found a greater LVM in patients with RA, regardless of age, blood pressure, and BMI. The mechanisms that make RA induce changes in the morphology of the left ventricle have not been well established; however, in this pathology, there is a significant increase of inflammatory cytokines such as TNF-*α*, which in animal models have demonstrated to be able to induce remodeling of the left ventricle and cardiomyocyte hypertrophy [36]. It is plausible that the intrinsic inflammatory status of EAT could play a major role in affecting LVM in patients with RA. The correlation between epicardial fat thickness and left ventricular mass has been described previously [37], and various mechanisms could explain this relation; among them, (a) increased EAT is associated with greater intramyocardial lipid content, which could provoke adverse structural and functional adaptations, including left ventricular hypertrophy [38], and (b) EAT can affect cardiac morphology through the local release of adipokines able to induce cardiac remodeling [39]. Furthermore, at the systemic level, EAT could induce insulin resistance, which would serve as an intermediary between visceral fat and left ventricular hypertrophy [40].

It has been explained that TNF-*α* inhibitors are associated with significant changes in the lipid profile, mainly with the increase of both total and HDL cholesterol; however, these changes have been observed only in the good respondents to the treatment, which suggests that the lowering of the inflammation and not the specific treatment is the reason for such lipid modifications [41]. Our study revealed that the group treated with TNF-*α* inhibitors showed lower plasma levels for total cholesterol, LDL-C, triglycerides, and non-HDL-C and Tg/HDL-C ratio and a higher HDL-C plasma concentration than the group treated with nonbiological DMARDs, but this difference was not significant. It is worth noticing that there were no significant differences in glucose levels or in either SBP or DBP between the groups, partly because the selected patients did not suffer from diabetes mellitus or high blood pressure.

Although our study provides findings of absolute novelty, we recognize some limitations. First of all, the sample size was relatively small but sufficient to detect a statistically significant difference among study groups. Secondly, given the cross-sectional design no conclusions on the effect of role of DMARDs on EAT can be drawn. Thirdly, the lack of another visceral fat imaging prevents us from drawing final conclusion on the superiority of EAT over other markers of visceral adiposity in the clinical setting of RA. However, a number of studies have previously shown the advantages of ultrasound as an easy and no invasive measure visceral fat in other clinical scenarios [14].

Further prospective studies with larger samples are necessary to confirm these findings and evaluate if echocardiographic EAT thickness can provide additional information for cardiometabolic risk stratification of RA patients.

## Figures and Tables

**Table 1:** Anthropometric and clinical variables of the control group, the group of subjects with RA treated with nonbiological DMARDs, and the group of RA subjects treated with biological DMARDs.

Variable	Control group *n* = 16	RA nonbiological DMARDs *n* = 16	RA biological DMARDs *n* = 18
Age (years)	51.81 ± 9.75	51.31 ± 6.70	52.05 ± 8.26
Weight (Kg)	75.46 ± 22.85	69.86 ± 14.18	70.82 ± 19.96
Height (m)	1.59 ± 0.05	1.56 ± 0.07	1.58 ± 0.05
BMI (Kg/m^2^)	29.48 ± 7.35	28.65 ± 7.09	28.20 ± 8.19
WC (cm)	89.84 ± 17.74	91.31 ± 15.89	88.41 ± 27.20
SBP (mmHg)	117.50 ± 11.25	121.00 ± 7.10	122.11 ± 8.82
DBP (mmHg)	75.93 ± 9.86	79.18 ± 5.23	78.72 ± 4.72

Continuous variables are presented as *X* ± SD.

BMI: body mass index, WC: waist circumference, SBP: systolic blood pressure, and DBP: diastolic blood pressure.

**Table 2:** Biochemical variables and left ventricular mass of the control group, the group of RA subjects treated with nonbiological DMARDs, and those treated with biological DMARDs.

Variable	Control group *n* = 16	RA nonbiological DMARDs *n* = 16	RA biological DMARDs *n* = 18
Glucose (mg/dL)	94.87 ± 8.87	91.43 ± 16.07	86.22 ± 10.37
Basal insulin (mU/mL)	12.25 ± 6.85	13.09 ± 7.92	13.32 ± 6.32
Total cholesterol (mg/dL)	197.43 ± 50.23	212.37 ± 48.54	197.55 ± 54.70
HDL-C (mg/dL)	50.53 ± 14.52	51.91 ± 15.41	55.49 ± 13.47
LDL-C (mg/dL)	121.24 ± 47.83	130.81 ± 36.78	118.55 ± 41.86
Non-HDL-C (mg/dL)	146.90 ± 44.60	160.45 ± 47.45	142.06 ± 49.90
Triglycerides (mg/dL)	140.62 ± 54.33	148.18 ± 84.75	117.66 ± 64.08
Tg/HDL-C ratio	2.90 ± 1.19	3.11 ± 2.26	2.24 ± 1.49
hs-CRP (mg/L)	1.61 ± 0.82	2.79 ± 1.51^*^	2.76 ± 1.94^*^
LVM (g/m^2^)	84.62 ± 26.07	130.44 ± 25.77^**^	120.94 ± 29.53^**^

Continuous variables are presented as X ± SD.

HDL-C: high-density lipoprotein, LDL-C: low-density lipoprotein, Tg: triglycerides, hs-CRP: high-sensitivity C-reactive protein, and LVM: left ventricular mass. ^*^ *P* = 0.033 versus control group. ^**^ *P* = 0.0001 versus control group.

**Table 3:** Acute phase reactants and rheumatoid arthritis activity indicators in the group of subjects treated with nonbiological DMARDs and those treated with biological DMARDs.

Variable	RA nonbiological DMARDs *n* = 16	RA biological DMARDs *n* = 18
ESR (mm/hour)	49.62 ± 25.68	38.94 ± 20.87
RF (mg/dL)	216.79 ± 266.95	190.00 ± 262.14
DAS 28	5.10 ± 1.36	4.98 ± 1.47

Continuous variables are presented as *X* ± SD.

ESR: erythrocyte sedimentation rate and RF: rheumatoid factor.

**Table 4:** Multiple linear regression analysis of the variables related to epicardial fat thickness as a dependent variable.

Independent variables	*P* value	
Age (years)	0.798	
BMI (kg/m^2^)	0.966	
WC (cm)	0.268	
hs-CRP (mg/L)	0.191	
LVM (g/m^2^)	0.151	
Control patient	0.0001	*R* ^2^: 0.595
		Coef. *β*: 2.892
		CI: 95%: 1.513–4.271

BMI: body mass index, WC: waist circumference, hs-CRP: high-sensitivity C-reactive protein, and LVM: left ventricular mass.
